# Supplementary figures and images for: Impact of two neighbouring ribosomal protein clusters on biogenesis factor binding and assembly of yeast late small ribosomal subunit precursors
Source: PLoS One. 2019 Jan 17;14(1):e0203415. doi: 10.1371/journal.pone.0203415 (PMC6336269; doi:10.1371/journal.pone.0203415)

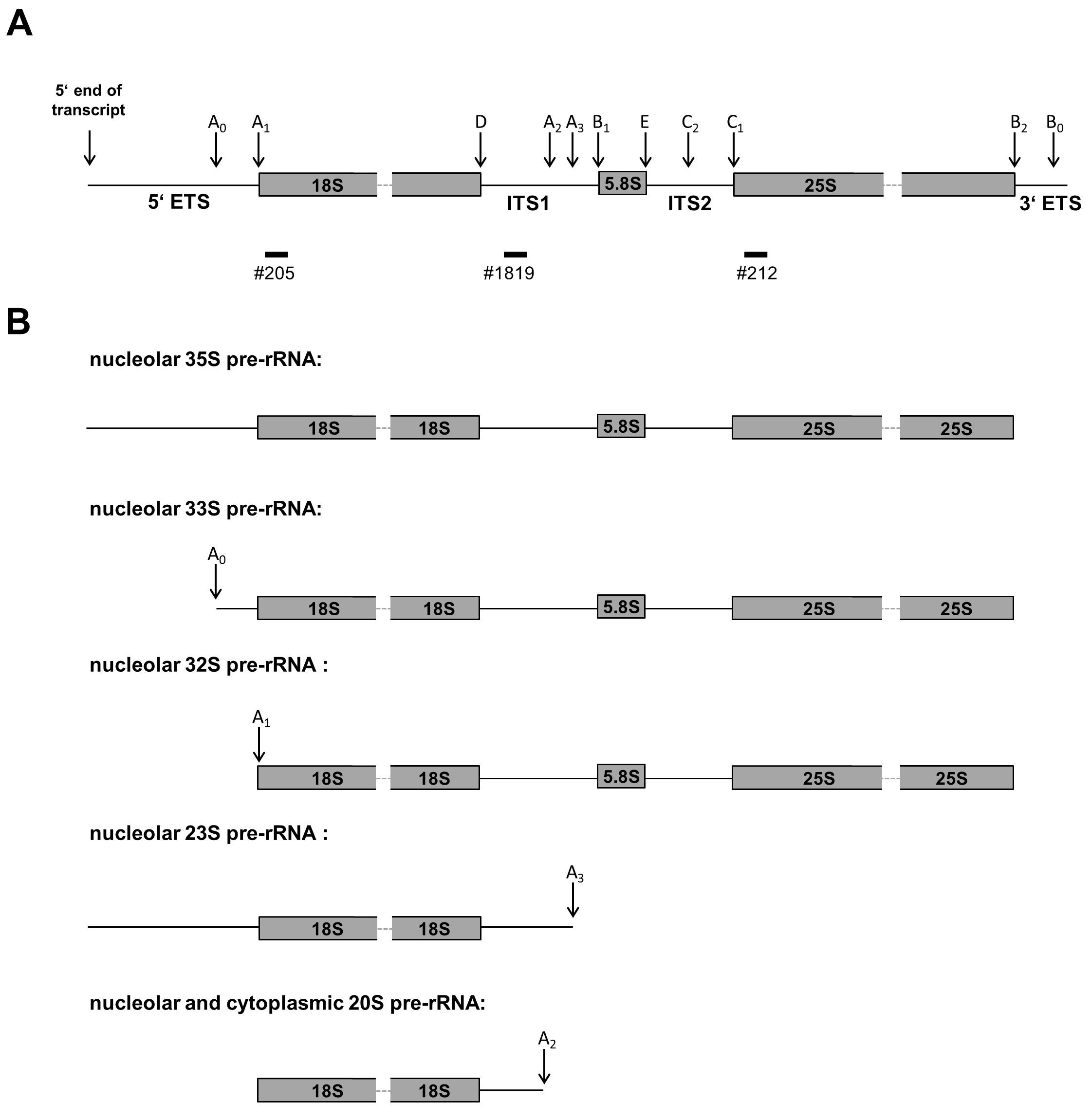

Supplement: S1 Fig — In (A) the primary transcript of 35S rRNA genes is schematically represented with 18S, 5.8S and 25S rRNA regions shown as grey boxes (not drawn to scale) and with external and internal spacers shown as continuous thin black line. Positions of major processing sites are highlighted by arrows and binding sites of oligonucleotides used in this study are indicated by black bars. In (B) the major steady state SSU pre-rRNA populations detected in S. cerevisiae are shown which result from co- or posttranscriptional processing events. (TIF) [file pone.0203415.s001.tif]

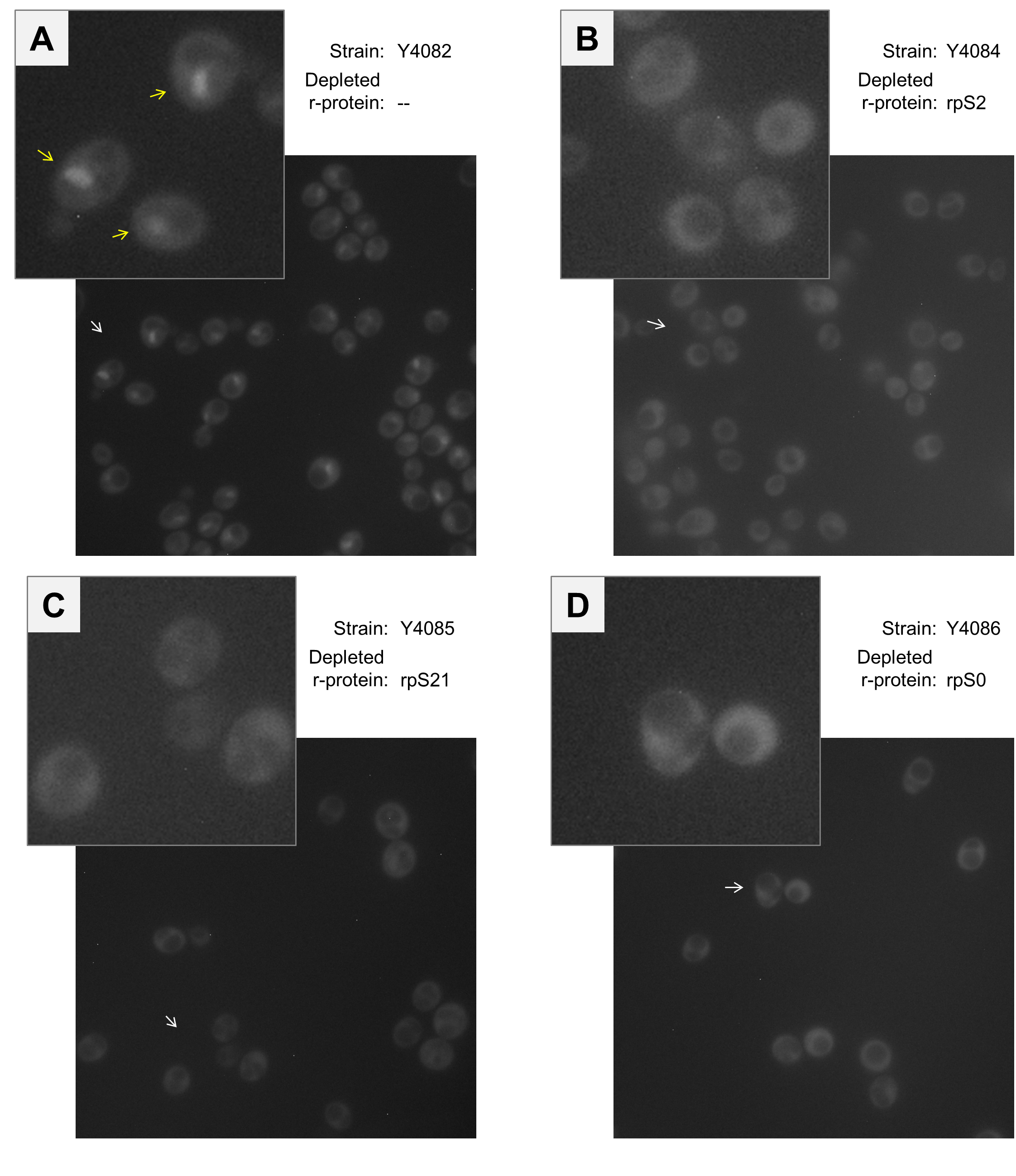

Supplement: S2 Fig — The indicated yeast strains were cultivated for four hours in glucose containing medium (YPD) to shut down expression of either rpS0, rpS2, rpS21 or no r-protein. Cellular localization of GFP tagged Rrp12 as visualized by fluorescence microscopy (see Materials and Methods) is shown in (A)—(D). Areas indicated with white arrows are shown enlarged and with increased contrast in the upper left inserts in (A)-(D). In the enlarged insert in (A) concentrated crescent shaped fluorescence signal, which is characteristic for the yeast nucleolus, is highlighted by yellow arrows. (TIF) [file pone.0203415.s002.tif]
