# Supplementary material for: Impact of two neighbouring ribosomal protein clusters on biogenesis factor binding and assembly of yeast late small ribosomal subunit precursors
Source: PLoS One. 2019 Jan 17;14(1):e0203415. doi: 10.1371/journal.pone.0203415 (PMC6336269; doi:10.1371/journal.pone.0203415)
Supplement: S1 File — (PDF) [file pone.0203415.s003.pdf]

oligo id	name	sequence
O205	o2-18S	CATGGCTTAATCTTTGAGAC
O212	o9-25S	CTCCGCTTATTGATATGC
O416	YCplac-Flag-5'	GATCATGGATTACAAGGATGACGACGATAAGGGTACCG
O417	YCplac-Flag-3'	GATCCGGTACCCTTATCGTCGTCATCCTTGTAATCCAT
O515	DIM2_S3	TATGGGAACTTACGTACCGTTGCATCTAGATTAAAAGAACGCTACCGTACGCTGCAGGTCGAC
O520	RRP12_pBS_DO	TTTTCTCCAGGTGTGTAATTAGCCATATTGCTCAGTTTCAATCTTTACGACTCACTATAGGG
O521	RRP12_pBS_UP	AAACATAATAAGAAAGGTCCAAAGTTCAAATCTAGAAAAAAATTATCCATGGAAAAGAGAAG
O528	9myc-ende-BglII	TTTTTTAGATCTTTTTTAGCTAGTGGATCC
O547	TSR1_S3	TACAAACGTATGTGGCCCATGCCTTCGTTACCTTGGAATGGTATGCGTACGCTGCAGGTCGAC
O548	TSR1_S2b	CGAACGGAACAGGTATCGACTTACCTTTATCAACAGTATCGTTGATACATCGATGAATTCGAGCTCG
O567	UTP7_S2	ATACATACATAATCATTTAAGTTTTTTTTTTAAAGATATTTCGATATCGATGAATTCGAGCTCG
O568	UTP7_S3	AGAAGACCACAAGGATGTCATCGAAGAGGCATTGAGCAGATTCGGCCGTACGCTGCAGGTCGAC
O1819	ext_ITS1_2	GTAAAAGCTCTCATGCTCTTGCC
O2316	RIO2_pBS_UP	GGTGTTGAAAATCTAAAAATGGATAAACTAGGAAACTATATACTAGAGTCCATGGAAAAGAGAAG
O2317	RIO2_pBS_DO	GGATAACAACTTGATTATTTGCGGCCATTTATGCAGTCGTCTAAACTAAATACGACTCACTATAGGG
O3877	SLX9_pBS_UP	AATCCATTTGGCGCCTTAAGAGAGGTTATCAAGCTGCAAAAACAATCCATGGAAAAGAGAAG
O3878	SLX9_pBS_DO	TATATATTACACTGGCAAAAATTGTTATGCTATGCTATTTAATGTTACGACTCACTATAGGG
