# Supplementary material for: Impact of two neighbouring ribosomal protein clusters on biogenesis factor binding and assembly of yeast late small ribosomal subunit precursors
Source: PLoS One. 2019 Jan 17;14(1):e0203415. doi: 10.1371/journal.pone.0203415 (PMC6336269; doi:10.1371/journal.pone.0203415)
Supplement: S2 File — (PDF) [file pone.0203415.s004.pdf]

| plasmid id | name | origin |
|---|---|---|
| | pBS1539 | [59] |
| | pym3 | [60] |
| | pAG32 | [63] |
| K230 | YCplac111pGAL | [43] |
| K231 | YCplac111pGAL_FLAG | A hybrid of Oligos O416 and O417 was 5' phosphomodified and subsequently ligated with BamHI digested plasmid K230 |
| K2378 | pBS1539-eGFP-3xHA | a synthetic gene fragment (tccatggaaaagagaagcctcggcgcctgtgagctcggagcaggtgctggtgctggtgctggagcaatgagcaagggcgaggagctgttcaccggggtggtgcccatcctggtcgagctggacggcgacgtaaacggccacaagttcagcgtgtccggcgagggcgagggcgatgccacctacggcaagctgaccctgaagttcatctgcaccaccggcaagctgcccgtgccctggcccaccctcgtgaccaccctgacctacggcgtgcagtgcttcagccgctaccccgaccacatgaagcagcacgacttcttcaagtccgccatgcccgaaggctacgtccaggagcgcaccatcttcttcaaggacgacggcaactacaagacccgcgccgaggtgaagttcgagggcgacaccctggtgaaccgcatcgagctgaagggcatcgacttcaaggaggacggcaacatcctggggcacaagctggagtacaactacaacagccacaacgtctatatcatggccgacaagcagaagaacggcatcaaggtgaacttcaagatccgccacaacatcgaggacggcagcgtgcagctcgccgaccactaccagcagaacacccccatcggcgacggccccgtgctgctgcccgacaaccactacctgagcacccagtccgccctgagcaaagaccccaacgagaagcgcgatcacatggtcctgctggagttcgtgaccgccgccgggatcactctcggcatggacgagctgtacaagagatctgagctcatcttttacccatacgatgttcctgactatgcgggctatccctatgacgtcccggactatgcaggatcctatccatatgacgttccagattacgctgctcagtgctagaagctt) was cloned into vector pBS1539 using restriction enzymes NcoI and HindIII |
| K251 | Ycplac111pGAL_RPS0 | [43] |
| K252 | Ycplac111-pGAL_FLAG_RPS0B | The RPS0 coding sequence was subcloned from K251 into K231 using restrictrion enzymes BamHI and PstI |
| K255 | Ycplac111pGAL_RPS2 | [43] |
| K256 | Ycplac111pGAL_FLAG_RPS2 | The RPS2 coding sequence was subcloned from K255 into K231 using restrictrion enzymes BamHI and PstI |
| K257 | Ycplac111pGAL_RPS3 | [43] |
| K258 | Ycplac111pGAL_FLAG_RPS3 | The RPS3 coding sequence was subcloned from K257 into K231 using restrictrion enzymes BamHI and PstI |
| K274 | Ycplac111pGAL_RPS20 | [43] |
| K275 | Ycplac111pGAL_FLAG_RPS20 | The RPS20 coding sequence was subcloned from K275 into K231 using restrictrion enzymes BamHI and PstI |
| K354 | 9xmyc-hphMX4 | A part of vector pym6 was amplified by PCR using primers O515 and O528 and the resulting fragment was cloned into plasmid pAG32 using restriction enzymes SalI and BglII |
