# Supplementary material for: Impact of two neighbouring ribosomal protein clusters on biogenesis factor binding and assembly of yeast late small ribosomal subunit precursors
Source: PLoS One. 2019 Jan 17;14(1):e0203415. doi: 10.1371/journal.pone.0203415 (PMC6336269; doi:10.1371/journal.pone.0203415)
Supplement: S3 File — (PDF) [file pone.0203415.s005.pdf]

strain id	name	genotype	plasmids propagated	mating type	source
Euroscarf Y07202		BY4741, *ura3Δ0, leu2Δ0, his3Δ1, met15Δ0, YDR007w::kanMX4*		a	Euroscarf
Euroscarf Y10305		BY4742, *ura3Δ0, leu2Δ0, his3Δ1, lys2Δ0, YEL063c::kanMX4*		alpha	Euroscarf
Euroscarf Y02384		BY4741, *ura3Δ0, leu2Δ0, his3Δ1, met15Δ0, YOR128c::kanMX4*		a	Euroscarf
Y180	RPS0-Shuffle	*his3-1, leu2-0, ura3-0, lys2-0, met15-0, YLR048w::kanMX4, YGR214w::HIS3*	Ycplac33_RPS0B	alpha	[43]
Y186	RPS2-Shuffle	*his3-1, leu2-0, ura3-0, met15-0 lys2-0, YGL123w::kanMX4*	Ycplac33_RPS2	alpha	[43]
Y193	RPS20-Shuffle	*his3-1, leu2-0, ura3-0, met15-0, YHL015w::kanMX4*	Ycplac33_RPS20	alpha	[43]
Y268	RPS3-Shuffle	*his3-1, leu2-0, ura3-0, met15-0, lys2-0, YNL178w::kanMX4*	Ycplac33_RPS3	a	[43]
Y271	pGAL_FLAG_RPS0	*his3-1, leu2-0, ura3-0, lys2-0, met15-0, YLR048w::kanMX4, YGR214w::HIS3*	Ycplac111pGAL_FLAG_RPS0 (K252)	alpha	Derivative of Y180, transformed with plasmid K252, selection on 5-fluoroorotic acid containing plates
Y282	RS-alpha3	*his3-1, leu2-0, ura3-0, lys2-0, trp1::hisG, can1::hisG, ade2::hisG*		alpha	The *kanMX4* deletion marker in the Euroscarf TRP1 deletion strain Y07202 was replaced by a *hisG* cassette using linearized plasmid M4786 [61]. The resulting strain was crossed with a derivative of Euroscarf strain Y10305 in which the *kanMX4* marker used for *CAN1* deletion had been replaced by a *hisG-URA3-hisG* cassette [61]. Uracil, tryptophan, lysin and methionin prototroph diploids were selected. Random spore analysis was then performed to select for haploid cells which were canavanin resistant and tryptophan auxotroph. The resulting (*Mat a, trp1::hisG, can1::hisG-URA3-hisG)* strain was crossed with a derivative of Euroscarf strain Y02384 in which the *kanMX4* marker used for *ADE2* deletion had been replaced by a *hisG* cassette [61]. Random spore analysis was performed to select for red coloured, canavanin resistant, uracil prototroph and tryptophan auxotroph haploid clones. Finally, the URA3 marker in the resulting strains was recycled according to Voth et al.
Y283	BY-a20	*his3-1, leu2-0, ura3-0, lys2-0, trp1::hisG, can1::hisG, ade2::hisG*		a	See Y282
Y286	pGAL_RPS2	*his3-1, leu2-0, ura3-0, met15-0, lys2-0, YGL123w::kanMX4*	Ycplac111pGAL_RPS2	alpha	[43]
Y317	pGAL_FLAG_RPS3	*his3-1, leu2-0, ura3-0, met15-0, lys2-0, YNL178w::kanMX4*	Ycplac111pGAL_FLAG_RPS3 (K258)	a	Derivative of Y268, transformed with plasmid K258, selection on 5-fluoroorotic acid containing plates
Y320	pGAL_FLAG_RPS20	*his3-1, leu2-0, ura3-0, met15-0, YHL015w::kanMX4*	Ycplac111pGAL_FLAG_RPS20 (K275)	alpha	Derivative of Y193, transformed with plasmid K275, selection on 5-fluoroorotic acid containing plates
Y327	pGAL_RPS3	*his3-1, leu2-0, ura3-0, met15-0, lys2-0, YNL178w::kanMX4*	Ycplac111pGAL_RPS3	a	[43]
Y335	pGAL_FLAG_RPS2	*his3-1, leu2-0, ura3-0, met15-0, lys2-0, YGL123w::kanMX4*	Ycplac111pGAL_FLAG_RPS2 (K256)	alpha	Derivative of Y186, transformed with plasmid K256, selection on 5-fluoroorotic acid containing plates
Y346	UTP7_6xHA	*his3-1, leu2-0, ura3-0, lys2-0, trp1::hisG, can1::hisG, ade2::hisG, UTP7::6xHA-klTRP1*		alpha	Derivative of strain Y282, cassette for homologous recombination at the UTP7 locus was amplified from plasmid pYM3 using oligos O567 and O568
Y360	TSR1_9xMyc	*his3-1, leu2-0, ura3-0, met15-0, TSR1::9xMyc-hphMX4*		a	Derivative of BY4741, cassette for homologous recombination at the TSR1 locus was amplified from plasmid K354 using oligos O547 and O548
Y391	RS-UTP7_6xHA-TSR1_9xMyc	*his3-1, leu2-0, ura3-0, trp1::hisG, can1::hisG, ade2::hisG, UTP7::6xHA-klTRP1, TSR1::9xMyc-hphMX4*		alpha	Strains Y346 and Y360 were crossed, haploides expressing myc tagged TSR1 and HA tagged Utp7 were selected after random spore analyses
Y401	RS-UTP7_6xHA-TSR1_9xMyc	*his3-1, leu2-0, ura3-0, trp1::hisG, can1::hisG, ade2::hisG, UTP7::6xHA-klTRP1, TSR1::9xMyc-hphMX4*		a	Strains Y346 and Y360 were crossed, haploides expressing myc tagged TSR1 and HA tagged Utp7 were selected after random spore analyses
Y408	UTP7_6xHA-TSR1_9xMyc-RIO2_TAP	*his3-1, leu2-0, ura3-0, trp1::hisG, can1::hisG, ade2::hisG, UTP7::6xHA-klTRP1, TSR1::9xMyc-hphMX4, RIO2::TAP-klURA3*		alpha	Derivative of strain Y391, cassette for homologous recombination at the RIO2 locus was amplified from plasmid pBS1539 using oligos O2316 and O2317
Y409	UTP7_6xHA TSR1_9xMyc-RIO2_TAP	*his3-1, leu2-0, ura3-0, trp1::hisG, can1::hisG, ade2::hisG, UTP7::6xHA-klTRP1, TSR1::9xMyc-hphMX4, RIO2::TAP-klURA3*		a	Derivative of strain Y401, cassette for homologous recombination at the RIO2 locus was amplified from plasmid pBS1539 using oligos O2316 and O2317
Y427	pGAL_FLAG_RPS3 RIO2_TAP	*his3-1, leu2-0, ura3-0, can1::hisG, ade2::hisG, TSR1-9xMyc::hphMX4, RIO2::TAP-klURA3, YNL178w::kanMX4*	Ycplac111pGAL_FLAG_RPS3 (K258)	n.d.	Strains Y317 and Y408 were crossed, haploide clones expressing TAP tagged Rio2 were selected after random spore analyses
Y428	pGAL_FLAG_RPS20 RIO2_TAP	*his3-1, leu2-0, ura3-0, can1::hisG, UTP7::6xHA-klTRP1, TSR1::9xMyc-hphMX4, RIO2::TAP-klURA3, YHL015w::kanMX4*	Ycplac111pGAL_FLAG_RPS20 (K275)	n.d.	Strains Y320 and Y409 were crossed, haploide clones expressing TAP tagged Rio2 were selected after random spore analyses
Y430	pGAL_FLAG_RPS2 RIO2_TAP	*his3-1, leu2-0, ura3-0, can1::hisG, ade2::hisG, UTP7::6xHA-klTRP1, TSR1::9xMyc-hphMX4, RIO2::TAP-klURA3, YGL123w::kanMX4*	Ycplac111pGAL_FLAG_RPS2 (K256)	n.d.	Strains Y335 and Y409 were crossed, haploide clones expressing TAP tagged Rio2 were selected after random spore analyses
Y446	pGAL_FLAG_RPS0 RIO2_TAP	*his3-1, leu2-0, ura3-0, lys2-0, met15-0, YLR048w::kanMX4, YGR214w::HIS3, RIO2::TAP-klURA3*	Ycplac111pGAL_FLAG_RPS0 (K252)	alpha	Derivative of strain Y271, cassette for homologous recombination at the RIO2 locus was amplified from plasmid pBS1539 using oligos O2316 and O2317
Y801	pGAL_RPS21	*his3-1, leu2-0, ura3-0, YKR057w::kanMX4, YJL136c::kanMX4*	Ycplac111pGAL_RPS21A	n.d.	[52]
Y1236	pGAL_RPS29	*his3-1, leu2-0, ura3-0, YDL061c::kanMX4, YLR388w::HIS3*	Ycplac111pGAL_RPS29B	n.d.	[52]
Y2704	pGal_RPS21-Rio2_TAP	*his3-1, leu2-0, ura3-0, YKR057w::kanMX4, YJL136c::kanMX4, RIO2::TAP-klURA3*	Ycplac111pGAL_RPS21A	n.d.	Derivative of strain Y801, cassette for homologous recombination at the RIO2 locus was amplified from plasmid pBS1539 using oligos O2316 and O2317
Y2705	pGAL_RPS29-Rio2_TAP	*his3-1, leu2-0, ura3-0, YDL061c::kanMX4, YLR388w::HIS3, RIO2::TAP-klURA3*	Ycplac111pGAL_RPS29B	n.d.	Derivative of strain Y1236, cassette for homologous recombination at the RIO2 locus was amplified from plasmid pBS1539 using oligos O2316 and O2317
Y2749	Rio2_TAP	*his3-1, leu2-0, ura3-0, lys2-0, RIO2::TAP-klURA3*		alpha	Derivative of strain BY4742, cassette for homologous recombination at the RIO2 locus was amplified from plasmid pBS1539 using oligos O2316 and O2317
Y3146	Slx9_TAP	*his3-1, leu2-0, ura3-0, met15-0, SLX9::TAP-klURA3*		a	Derivative of strain BY4741, cassette for homologous recombination at the SLX9 locus was amplified from plasmid pBS1539 using oligos O3877 and O3878
Y3149	pGAL_RPS3-Slx9_TAP	*his3-1, leu2-0, ura3-0, met15-0, lys2-0, YNL178w::kanMX4, SLX9::TAP-klURA3*	Ycplac111pGAL_RPS3	a	Derivative of strain Y327, cassette for homologous recombination at the SLX9 locus was amplified from plasmid pBS1539 using oligos O3877 and O3878
Y3151	pGAL_RPS2-Slx9_TAP	*his3-1, leu2-0, ura3-0, met15-0, lys2-0, YGL123w::kanMX4, SLX9::TAP-klURA3*	YCplac111pGAL_RPS2	alpha	Derivative of strainY286, cassette for homologous recombination at the SLX9 locus was amplified from plasmid pBS1539 using oligos O3877 and O3878
Y3153	pGal_RPS21-Slx9_TAP	*his3-1, leu2-0, ura3-0, YKR057W::kanMX4, YJL136C::kanMX4, SLX9::TAP-klURA3*	Ycplac111pGAL_RPS21A	n.d.	Derivative of strain Y801, cassette for homologous recombination at the SLX9 locus was amplified from plasmid pBS1539 using oligos O3877 and O3878
Y4063	pGAL_FLAG_RPS0-Slx9_TAP	*his3-1, leu2-0, ura3-0, lys2-0, met15-0, YLR048w::kanMX4, YGR214w::HIS3, SLX9::TAP-klURA3*	Ycplac111pGAL_FLAG_RPS0B (K252)	alpha	Derivative of strain Y271, cassette for homologous recombination at the SLX9 locus was amplified from plasmid pBS1539 using oligos O3877 and O3878
Y4082	Rrp12_GFP_3xHA	*his3-1, leu2-0, ura3-0, met15-0, RRP12::GFP_3xHA-klURA3*		a	Derivative of strain BY4741, cassette for homologous recombination at the RRP12 locus was amplified from plasmid K2378 using oligos O520 and O521
Y4084	pGAL_RPS2-Rrp12_GFP_3xHA	*his3-1, leu2-0, ura3-0, met15-0, lys2-0, YGL123w::kanMX4, RRP12::GFP_3xHA-klURA3*	YCplac111pGAL_RPS2	alpha	Derivative of strain Y286, cassette for homologous recombination at the RRP12 locus was amplified from plasmid K2378 using oligos O520 and O521
Y4085	pGal_RPS21-Rrp12_GFP_3xHA	*his3-1, leu2-0, ura3-0, YKR057W::kanMX4, YJL136C::kanMX4, RRP12::GFP_3xHA-klURA3*	Ycplac111pGAL_RPS21A	n.d	Derivative of strain Y801, cassette for homologous recombination at the RRP12 locus was amplified from plasmid K2378 using oligos O520 and O521
Y4086	pGAL_FLAG_RPS0-Rrp12_GFP_3xHA	*his3-1, leu2-0, ura3-0, lys2-0, met15-0, YLR048w::kanMX4, YGR214w::HIS3, RRP12::GFP_3xHA-klURA3*	Ycplac111pGAL_FLAG_RPS0B (K252)	alpha	Derivative of strain Y271, cassette for homologous recombination at the RRP12 locus was amplified from plasmid K2378 using oligos O520 and O521
